# Interests and preferences regarding family planning self-care interventions: cross-sectional surveys with Kenyan and Nigerian women

**DOI:** 10.1080/26410397.2026.2681342

**Published:** 2026-06-01

**Authors:** Funmilola M. OlaOlorun, Fredrick Makumbi, Simon Peter Sebina Kibira, Elena Lebetkin, Ndola Prata, Philip Anglewicz, Peter Gichangi, Elizabeth Omoluabi, Aisha Siewe, Mary Thiongo, Musa Sani Zakirai, Aurélie Brunie

**Affiliations:** a Consultant, Evidence for Sustainable Human Development in Africa (EVIHDAF), Yaoundé, Cameroon; Associate Professor, Department of Community Medicine, College of Medicine, University of Ibadan, Ibadan, Nigeria.; b Professor, School of Public Health, Makerere University, Kampala, Uganda; c Senior Lecturer, School of Public Health, Makerere University, Kampala, Uganda; d Technical Advisor, FHI 360, Washington DC & North Carolina, USA; e Consultant, Evidence for Sustainable Human Development in Africa (EVIHDAF), Yaoundé, Cameroon; Professor in Residence of Maternal, Child and Adolescent Health, School of Public Health, University of California, Berkeley, USA; f Professor, Department of Population, Family & Reproductive Health, Johns Hopkins University, Baltimore, MD, USA; g Principal Investigator, International Center for Reproductive Health, Nairobi, Kenya; Deputy Vice-Chancellor, Academic, Research and Extension, Technical University of Mombasa, Mombasa, Kenya; Visiting Professor, Department of Public Health and Primary Care, Faculty of Medicine and Health Sciences, Ghent University, Ghent, Belgium; h Research Fellow, Department of Statistics and Population Studies, University of the Western Cape, Cape Town, South Africa; Executive Director, Center for Research Evaluation Resources and Development, Nigeria; i Associate Director for Data, Department of Population, Family & Reproductive Health, Johns Hopkins University, Baltimore, MD, USA; j Project Director, International Center for Reproductive Health, Nairobi, Kenya; k Co-Principal Investigator, Performance Monitoring for Action Project, Center for Research Evaluation Resources and Development, Ile-Ife, Nigeria; l Scientist, FHI 360, Washington DC & North Carolina, USA

**Keywords:** self-care, family planning preferences, oral contraceptive pills, emergency contraception, DMPA

## Abstract

Self-care interventions for family planning encourage women to play a central role in their own health care. This study assesses women’s interests and preferences related to mobile access to information and products amenable to self-administration, and examines factors associated with preferred place of access. We added a module to the female questionnaire of the cross-sectional Performance Monitoring for Action survey in Kenya (*n* = 9,489) and in two Nigerian states (Kano (*n* = 1,144) and Lagos (*n* = 1,426)) between November 2021 and January 2022. We analysed data on interests and preferences related to mobile access to information and various places of access for oral contraceptive pills, emergency contraception, and DMPA subcutaneous self-injection and fitted multinomial regression models to examine the factors associated with preferred place of access for each contraceptive product. Across sites, 69–85% of women were interested in accessing family planning and fertility information on their own. Among them, 88–90% were interested in receiving information via voice/text and 49–72% through social media. The preferred access point across products and sites was the health facility (56–82% of women), with some interest in other sources. Across sites, 71% or more of respondents said engaging with a provider was “very/somewhat important” when starting or while using all three methods. Multivariable analyses revealed differences in preferred place of access between subgroups of potential end users. Findings highlight opportunities for self-care interventions through health facilities and other access points and the need for multipronged tailored interventions that offer a range of touchpoints with the health system to enable choice.

## Plain language summary

Self-care encourages women to be at the centre of their own health care. The 2019 World Health Organization guideline on self-care interventions for sexual and reproductive health and rights has sparked renewed interest in self-care for family planning. However, we need evidence on women’s interests and preferences related to self-care interventions to guide country programmes. This study assesses women’s interests and preferences related to mobile access to family planning and fertility information, as well as contraceptive products that can be self-administered. We found that Kenyan and Nigerian women are interested in receiving information via voice or text message, and to a lesser extent via social media. The health facility remains the preferred place of access for products that can be self-administered, including contraceptive pills, emergency contraception and self-injection. But many women were also interested in accessing these products through drug shops and pharmacies, and some through home delivery. Most women reported that engaging with a provider when starting or while using these products was important. Recognising women’s rights to make and act on informed decisions about using contraception requires interventions that align with their interests and preferences. Our findings show that self-care options can improve women's access to the information and services they need. Digital interventions, drug shops, pharmacies and home delivery can increase choice and access. Adequate linkages to the health system should support this. Programme managers should consider ways to include self-care options alongside the health system.

## Résumé

Les interventions d’auto-prise en charge en matière de planification familiale encouragent les femmes à jouer un rôle central dans leur propre santé. Cette étude évalue les intérêts et les préférences des femmes concernant l’accès mobile aux informations et produits auto-administrables, et examine les facteurs associés au lieu d’accès préféré. Nous avons ajouté un module au questionnaire destiné aux femmes dans le cadre de l’enquête transversale de suivi de la performance pour l’action menée au Kenya (*n* = 9489) et dans deux États nigérians (Kano (*n* = 1144) et Lagos (*n* = 1426)) entre novembre 2021 et janvier 2022. Nous avons analysé les données relatives aux intérêts et aux préférences concernant l’accès mobile aux informations et les différents lieux d’accès aux pilules contraceptives orales, à la contraception d’urgence et aux auto-injections sous-cutanées d’AMPR, et nous avons utilisé des modèles de régression multinomiale pour examiner les facteurs associés au lieu d’accès préféré pour chaque produit contraceptif. Dans l’ensemble des sites, de 69 à 85% des femmes souhaitaient accéder elles-mêmes aux informations sur la planification familiale et la fécondité. Parmi elles, de 88 à 90% souhaitaient recevoir des informations par message vocal ou texte et de 49 à 72% par le biais des réseaux sociaux. Le point d’accès préféré, tous produits et sites confondus, était le centre de santé (de 56 à 82% des femmes), bien que d’autres sources aient suscité un certain intérêt. Dans l’ensemble des sites, 71% des répondantes ou plus ont déclaré que le contact avec un prestataire était « très important/assez important » au début ou pendant l’utilisation des trois méthodes. Les analyses multivariées ont révélé des différences dans le lieu d’accès préféré entre des sous-groupes d’utilisatrices finales potentielles. Ces résultats mettent en lumière les possibilités d’interventions d’auto-prise en charge par le biais des centres de santé et d’autres points d’accès, de même que la nécessité d’interventions personnalisées et pluridimensionnelles offrant un éventail de points de contact avec le système de santé afin de favoriser le choix.

## Resumen

Las intervenciones de autocuidado para la planificación familiar motivan a las mujeres a desempeñar un papel central en el cuidado de su salud. Este estudio evalúa los intereses y las preferencias de las mujeres relacionados con el acceso móvil a información y productos aptos para la autoadministración, y examina los factores asociados con el lugar de acceso preferido. Agregamos un módulo al cuestionario para mujeres de la encuesta transversal Monitoreo del desempeño para la acción en Kenia (*n* = 9,489) y en dos estados nigerianos (Kano (*n* = 1,144) y Lagos (*n* = 1,426)) entre noviembre de 2021 y enero de 2022. Analizamos los datos sobre intereses y preferencias relacionados con el acceso móvil a información y diversos lugares de acceso a píldoras anticonceptivas orales, anticoncepción de emergencia y la inyección anticonceptiva subcutánea AMPD, y ajustamos los modelos de regresión multinomial para examinar los factores asociados con el lugar de acceso preferido para cada producto anticonceptivo. En todos los sitios, entre el 69% y el 85% de las mujeres estaban interesadas en acceder a la información sobre planificación familiar y fertilidad por su cuenta. Entre ellas, del 88% al 90% estaban interesadas en recibir información vía voz/texto y del 49% al 72% por medio de redes sociales. El lugar de acceso preferido para todos los productos y en todos los sitios era el centro de salud (del 56% al 82% de las mujeres), con cierto interés en otras fuentes. En todos los sitios, el 71% o más de las mujeres encuestadas dijeron que interactuar con un prestador de servicios era “muy/un tanto importante” al iniciar los tres métodos o durante su uso. Los análisis multivariables revelaron diferencias en el lugar de acceso preferido entre subgrupos de posibles usuarias finales. Los hallazgos destacan oportunidades para intervenciones de autocuidado por medio de centros de salud y otros lugares de acceso, así como la necesidad de intervenciones adaptadas multifacéticas que ofrezcan una variedad de puntos de contacto con el sistema de salud para facilitar la elección.

## Introduction

Self-care is a modern name for a very old concept. Long before formal health systems existed, people managed their own health using herbal remedies, traditional practices, and knowledge passed down through generations. Today, self-care is shaped by advances in medicine, technology, and health information. Thus, the term self-care may be relatively new, but the practice is as old as humanity itself. The World Health Organization (WHO) defines self-care as the ability of individuals, families and communities to promote health, prevent disease and maintain health with or without the support of a healthcare provider.^1^ The WHO further defines self-care interventions, which include “evidence-based, quality drugs, devices, diagnostics and/or digital technologies which can be provided fully or partially outside of formal health services and can be used with or without the direct supervision of healthcare personnel” as tools to support self-care.^2^ In respect of family planning (FP), the 2022 revised version of the WHO guideline and the global values and preferences survey that preceded the guideline’s development underscore over-the-counter provision of contraceptive methods that women may be able to self-administer – including oral contraceptive pills (OCPs), emergency contraceptives (EC), self-injection of subcutaneous depot medroxyprogesterone acetate (DMPA-SC), the contraceptive patch, contraceptive vaginal ring, and barrier methods including male and female condoms, diaphragm, and the cervical cap – as well as information that can be found online or on mobile apps.^1,3–5^

Family planning is just one of nine elements of sexual and reproductive health and rights (SRHR). 2019 estimates highlight a gap in meeting the sexual and reproductive health (SRH) needs of women in low- and middle-income countries (LMICs), where 218 million women of reproductive age have an unmet need for FP.^6^ Additionally, the aftermath of COVID-19 has compounded existing vulnerabilities in health systems. For example, there is a growing shortage of health workers, with a projected global gap of 10 million by 2030, largely concentrated in LMICs.^7,8^ In this context, self-care is seen as an important part of solutions for protecting women’s rights and ensuring access to universal health coverage.^1^ Self-care interventions may provide greater convenience, reduce costs, empower users or be better aligned with people’s choices. Alternatively, people may turn to them to overcome health worker shortages and other health system challenges like poor quality of care, stigma, or lack of access.^1,9^

A systematic review published in 2022 looked at the relationship between reproductive empowerment (individual agency, partner negotiation, partner violence) and different aspects of contraceptive self-care (access, acceptability, use or intention to use).^10^ In all, 3219 studies published between 2010 and 2020 were identified, but only 37 were included in the qualitative synthesis. One study showed a favourable association between individual agency and access to OCPs. None of the studies included assessed the relationship between reproductive empowerment and accessibility to contraceptive self-care. Thirteen studies showed a favourable association between individual agency, partner negotiation, or partner violence and use of one or more contraceptive self-care methods. Five studies showed a favourable association between individual agency or partner negotiation and intention to use one or more contraceptive self-care methods. The authors concluded that the causal relationships between reproductive empowerment and contraceptive self-care are poorly understood and warrant more research.^10^

Policy and practice efforts to advance family planning (FP) are moving towards a strong focus on women’s rights and choice, including the right to make and act on informed decisions about using contraception.^1^ This study applied WHO’s conceptual framework for self-care interventions^1^ to FP. The framework consists of four layers surrounding a core focus on person-centred health and well-being. The first layer includes the key principles of human rights, gender equality, and ethics. Self-care interventions for FP should be holistic and take the changing needs of individuals over the life course into account. The second layer of the framework recognises both traditional (e.g. health services, communities, traditional medicine and sociocultural practices, home) and non-traditional (e.g. pharmacies, digital technologies and platforms) places of access for FP information and services. An enabling environment that includes supportive laws and policies, and access to justice represents a third layer. Access to justice refers to access to a functional system through which redress can be sought whenever there is neglect or violation of rights in the context of self-care interventions.^1^ Finally, self-care interventions for FP must be considered within the context of an outermost layer of multiple accountability systems at the levels of the individual, government, public and private health sectors, and donors.^1^

Historically, in sub-Saharan Africa, health service providers have been considered by many to be the main source of FP information and services;^11^ however, other sources are rising in popularity. According to the 2024 Nigeria and 2022 Kenya Demographic and Health Surveys, most women obtained their most recent method from a public or private health facility (62% and 80% respectively). Looking more closely at common methods amenable to self-administration, pharmacies and drug shops were major sources of supply for OCPs and EC in Kenya and Lagos.^12,13^ Previous research in Nigeria and Kenya also shows that venues like drug shops are popular for obtaining short-acting methods of contraception, especially among young users.^14,15^

Digital platforms are also becoming popular for accessing SRH information,^16^ including FP, among adolescents in Rwanda because of their flexibility and ability to accommodate group messages in real time, as well as eliminating physical access barriers.^16,17^ Use of digital platforms for information allows for a “participatory culture” among users, with the appeal of bidirectional interactions.^18^ Adolescents in Sierra Leone have been reported to fill their FP and contraceptive knowledge gaps through a variety of digital platforms.^19^ A review of digital tools with FP content designed for individual users in low- and middle-income countries highlighted that the accuracy and comprehensiveness of the FP content in the digital tools reviewed varied greatly, with noticeable content gaps.^20^ Furthermore, researchers have documented ethical concerns regarding these information sources. James (2009)^21^ argues that people who interact with and through digital platforms are developing new mindsets in relation to “identity, privacy, ownership and authorship, credibility, and participation”. [For a detailed description of these ethical concerns, see James, 2009.^21^]

Like many sub-Saharan African (SSA) countries, Kenya and Nigeria aim to continue to decrease unmet need for contraception. Modern contraceptive prevalence among all women in Kenya is 46%,^22^ while in Nigeria it is 14% and 27% respectively for Kano and Lagos states where this study was conducted.^23^ Policymakers in both countries are increasingly interested in incorporating self-care strategies into FP policies and programmes.^24,25^ Because self-care is multifaceted and contextually specific, self-care programming needs to be designed with women’s interests and preferences in mind to lead to improved outcomes.

Most research on FP self-care interventions to date has been method-specific, notably most recently with a growing body of evidence on DMPA-SC.^26–34^ There is limited evidence about the interests and preferences of women in SSA that would provide decision-makers with information on the broader range of FP self-care interventions and how best to situate them. This study aims to generate evidence on women’s interests and preferences related to key FP self-care interventions. We focus on interventions outlined by the WHO, including digital information provision, and the three most commonly used methods amenable to self-administration (OCPs, EC, and DMPA-SC self-injection), other than condoms. We did not include condoms because they are already widely available, including outside of the traditional health system. Specific objectives are to: (1) describe what types of fertility and FP information women want and their interest in receiving it through digital interventions; (2) examine preferred places of access for products that women may be able to self-administer; (3) explore the perceived importance and benefits of engaging with healthcare providers within the context of FP products amenable to self-administration; and (4) examine factors associated with preferred place of access by product.

## Methods

### Study design and dates

We obtained cross-sectional data by adding a mini-module of 21 questions to the female questionnaire of the third phase of the mobile phone-assisted longitudinal surveys conducted by the Performance Monitoring for Action (PMA) project in Kenya and two Nigerian states (Kano and Lagos) between November 2021 and January 2022. PMA collected data from a nationally representative sample of households in Kenya and state-representative data in Nigeria using a two-stage stratified cluster design with urban-rural strata and with enumeration areas as clusters.^35^ All women ages 15–49 at sampled households were invited to answer a female questionnaire. Overall, 9,489 women in Kenya and 2,570 in Nigeria (1,144 in Kano and 1,426 in Lagos) completed the main PMA survey. The PMA project ran from March 1, 2019 to August 30, 2023, in Kenya and from March 1, 2019 to November 30, 2024, in Nigeria.

### Study settings and population

#### Kenya

Kenya has a population of 57 million distributed across 47 counties.^36^ In the 2022 nationally representative Demographic and Health Survey, 57% of currently married and 59% of sexually active unmarried Kenyan women reported using a modern contraceptive method. Injectables (20%) and implants (19%) were most frequently reported among married women while male condoms (20%) and injectables (16%) were preferred by sexually active unmarried women.^12^ Kenya’s national self-care guidelines state that promoting self-care for FP will play a role in Kenya’s pursuit of universal health coverage.^25^ The National Reproductive Health Policy notes the use of community-based distribution of contraceptive methods amenable to self-care as a strategy to reduce unmet need for family planning and to ensure access to reproductive health for all Kenyans.^37^

#### Nigeria

Nigeria, the most populous country in Africa, has an estimated population of 237 million people spread across 36 states and a Federal Capital Territory.^36^ This study was conducted in two of these states, Lagos in the more liberal south, and Kano in the more conservative north. In Lagos and Kano states, 31% and 11% of currently married women respectively reported modern contraceptive use. According to a 2023/2024 nationally representative survey, 15% of currently married Nigerian women used a modern contraceptive method with most of these women reporting use of implants (6%), injectables (4%) and male condoms (2%). On the other hand, 38% of sexually active unmarried Nigerian women used a modern contraceptive method, predominantly condoms (26%), but also OCPs (3%) and EC (3%).^38^ Nigeria’s National Guideline on Self-Care for Sexual, Reproductive, and Maternal Health endorses WHO’s recommendations for OCPs to be available without prescription as well as the distribution of DMPA-SC for self-injection. The document references other policy guidelines like the Essential Medicine List, the National Training Manual on Family Planning for Physicians and Nurses/Midwives, and National Guidelines for the Introduction and Scale -up of DMPA-SC SI that support these policy endorsements. The policy document also states that EC should be available over the counter without restriction.^24^

### Data collection

The mini-module was incorporated in the FP section of the survey questionnaire. During the interview, response bias was minimised by a careful explanation of why a second level of consent was required for the self-care mini-module. Questions in the mini-module covered women’s interest in information on a range of FP topics, interest in receiving this information digitally, desires and preferences for accessing OCPs, EC, and DMPA-SC self-injection from different places, and perceived importance and benefits of engaging or not with healthcare providers when starting or while using products amenable to self-administration.^39^

### Data analysis

We conducted descriptive analyses on women’s interest in accessing information through digital interventions, interest in accessing OCPs or EC and DMPA-SC through different channels, preferred place of access for each of the three products, and perceived importance and benefits of engaging or not with healthcare providers when obtaining or while using each of the three products. All available data were used for analysis. We also descriptively compared actual and preferred places of access for current users of OCPs, EC or injectables. Due to the small numbers of women who reported current use of DMPA-SC self-injection, we combined all injectable users together for analysis related to method use. We examined potential differences by age group for interest in access to information through digital interventions and perceived importance of engaging with a provider and we provide Chi-square test statistics.

We conducted multinomial regression analyses, separately for each site, to examine factors associated with preferred place of access by product. Even though Kano and Lagos are both states in Nigeria, the contexts and the sample of women differ on multiple characteristics; hence the decision to analyse the sites separately. We used outcome data from the mini-module and covariates from the main PMA female questionnaire. The dependent variable, preferred place of access for each product, was ascertained by asking where women would most like to obtain the product if they did not have to pay for it. Response options included health facility, community health worker, mobile clinic/ community event, drug shop or pharmacy, shop or market in the community, friend or relative, delivery to home, and other, with an additional option for the participant to say they were not interested in the method. The analytic sample for multivariable analyses excluded women who said they were not interested in the method. After examining the distribution of responses, options were recategorised into (1) health facility, (2) drug shop/pharmacy, and (3) other sources. We included the following categorical variables as covariates in the multinomial regression models: sociodemographic characteristics (education, marital status, age, religion, urban/rural residence, wealth quintile, urban/rural residence), fertility intentions, current modern contraceptive use, and contraceptive empowerment. Wealth and contraceptive empowerment quintiles or tertiles follow the PMA methodology. ^40–42^ The contraceptive empowerment sub scale of the Women and Girls Empowerment in SRH index has nine items, of which five items reflecting the existence of choice (motivations underlying contraceptive decision-making) and four items revealing the exercise of choice (ability to act on contraceptive goals). The subscale has been validated across seven SSA countries, including Kenya and Nigeria.^43^ We dropped two variables in the two Nigerian sites: religion due to a lack of variability across preferred place of access, and urban/rural residence because the Lagos sample was 100% urban, and we wanted to align variables across the two states.^40^ Sensitivity analysis conducted on data from Kano revealed that the exclusion of urban/rural residence from the multinomial model did not change the overall interpretation of the results. We assessed correlations between the covariates in the model and dropped the variable “parity” due to high correlation (r > 0.5) with age across all three sites. Sensitivity analysis conducted with parity rather than age yielded similar robust results across all three sites. We tested for multicollinearity before finalising the model specification for multinomial regression analysis. We accounted for the survey design by adjusting for clustering using the svyset command in Stata. All data analyses were conducted using Stata SE/15.1.

### Informed consent

One of the institutional review committees stated that the self-care mini-module would require a second level of electronically documented oral consent. This is as a result of the different focus of the module and thus the need to respect the rights of the women and give them an opportunity to opt out of the additional questions. All women provided written informed consent by signing or checking a box on the mobile device with the form. Assent was obtained from all unmarried minors (females under 18 years), after their parent/guardian had given their consent. For unmarried minors, both consent and assent were documented by a signature or check mark on the electronic form. All married females under 18 years of age were considered as emancipated minors, as per the study protocol, and enrolled in the study after providing informed consent. All interviewers were from the respective region or state and were able to explain the contents of the informed consent form in a language parents/guardians and minors could understand, and to answer any questions or concerns raised. There were a few parents/guardians of unmarried minors who were not willing to give their consent for minors to participate and this is reflected in the response rates.

### Ethical approvals

The following ethical review boards reviewed and approved the PMA survey: Kenya [Kenyatta National Hospital – University of Nairobi Ethics and Research Committee; P801/09/2019; Ethical approval dates: November 1, 2019; November 1, 2020; November 1, 2021]; Kano, Nigeria [Kano State Ministry of Health; AKTH/MAC/SUB/12A/P-3/V1/3751; Ethical approval dates: November 14, 2019; November 14, 2020; November 14, 2021]; Lagos, Nigeria [Lagos State University Teaching Hospital Health Research Ethics Committee; LREC/06/10/1276; November 4, 2019; November 20, 2020; September 20, 2021] and Johns Hopkins University [Johns Hopkins School of Public Health Institutional Review Board; IRB00014702]. All ethical approvals were renewed annually prior to each phase of data collection.

### Reflexivity

The authorship of this paper includes 12 authors from four countries (Kenya, Nigeria, Uganda and the United States) with backgrounds in epidemiology, medicine, social sciences, and statistics. Seven of the authors are female while five are male, with a mix of early, mid- and senior career researchers. Six of the authors are academics, while the other six work with non-governmental organisations or are non-academics working in a university setting. All authors have worked together previously on the PMA project or other studies.

## Results

**Figure 1.**
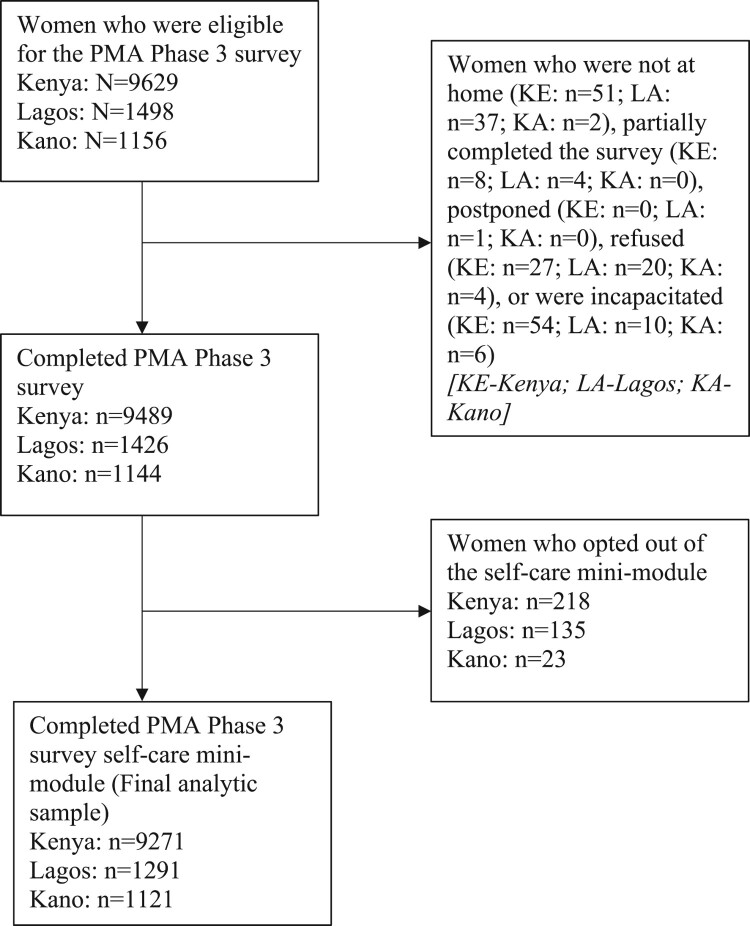
Flow chart showing the derivation of the analytic sample from the Performance Monitoring for Action Phase 3 study

Among women who completed the main PMA Phase 3 survey, 91% in Lagos and 98% in Kano and Kenya also completed the self-care mini-module. Response rates for the mini-module were 97% (*n* = 9,271), 98% (*n* = 1,121) and 89% (*n* = 1,291), respectively. The flow diagram showing the derivation of the analytic sample is depicted in Figure 1.

Most women participating in the mini-module were married or living with their partner, aged 25–49 years old and had at least one child (Table 1). While Lagos was exclusively urban (by design), 29% and 36% of participants in Kenya and Kano, respectively, lived in urban areas. Post-primary or higher education was reported by 90% of participants in Lagos, 56% in Kenya, and 39% in Kano. Overall, 38% of women in Lagos and 36% in Kano were in the highest wealth tertile; in Kenya, 37% were in the top two quintiles.

**Table 1.** Sociodemographic and economic characteristics of sample

	Kenya	Lagos, Nigeria	Kano, Nigeria
	Weighted % (*n* = 9271)	Weighted % (*n* = 1291)	Weighted % (*n* = 1121)
**Education**			
None/primary	44.3	10.4	61.3
Post-primary/secondary	41.0	53.8	33.5
College/Univ	14.7	35.8	5.3
**Marital status**			
Never married	35.6	37.0	24.4
Married/living with partner	54.4	57.2	71.7
Divorced/separated/widowed	10.1	5.8	3.9
**Age**			
15–24	41.0	30.7	42.8
25–49	59.0	69.3	57.2
**Religion**			
Catholic	15.2	8.7	0.2
Protestant	75.9	59.0	1.7
Islam	3.2	30.9	97.9
Unknown/other	5.6	1.4	0.2
**Parity**			
None	30.4	39.5	28.8
1–2	29.8	25.2	16.3
3–4	22.1	29.0	17.3
5+	17.7	6.3	37.6
**Geography/community**			
Rural/Urban residence		** **	** **
Rural	71.2	–	64.2
Urban	28.8	100	35.8
Wealth quintile/tertile*			
1. Lowest quintile/tertile	19.6	27.6	30.1
2. Lower quintile	23.8	–	–
3. Middle quintile/tertile	19.9	34.5	34.1
4. Higher quintile	18.7	–	–
5. Highest quintile/tertile	18.1	37.8	35.8
**Contraceptive use status**			
Current use of any modern method	46.0	27.0	13.9
**Desire for children**			
Undecided/ do not know	2.2	4.9	17.1
Have a/another child	62.0	65.1	62.7
Prefer no children/no more	34.2	29.6	15.9
Says she can't be pregnant	1.6	0.3	4.31
**Tertiles of contraceptive empowerment**			
Lower	29.0	34.2	35.7
Middle	35.4	15.4	22.4
Higher	35.6	50.4	42.0

*Calculated quintiles in Kenya and tertiles in Lagos and Kano due to the small sample size.

Altogether, 46% of mini-module participants in Kenya, 27% in Lagos, and 14% in Kano were using a modern method (Table 1). Around two-thirds of women across sites wanted to have (more) children. Half of participants in Lagos, 42% in Kano and 36% in Kenya were in the highest contraceptive empowerment tertile.

### Interest in access to information through digital interventions

#### Kenya

Eight out of ten women in Kenya were interested in accessing information on the FP-related topics proposed without consulting a healthcare provider at a health facility (see Supplementary Table 1a). FP-related topics included managing contraceptive-induced menstrual changes, managing side effects, identifying fertile days, confirming pregnancy, and assessing return to fertility postpartum. Most Kenyan women were interested in receiving information on any of these topics via voice/text and just over half through social media. Women aged 15–24 years preferred social media while women aged 25–49 years preferred voice/text messages.

#### Lagos

Seven out of ten women in Lagos were interested in receiving information on the FP-related topics proposed without having to access or speak with a healthcare provider at a health facility (see Supplementary Table 1b). Most women in Lagos were interested in voice/text messages and almost three quarters in social media. Women aged 15–24 years preferred social media while women aged 25–49 years preferred voice/text messages.

#### Kano

Eight out of ten women in Kano were interested in receiving information on the FP-related topics proposed without having to access or speak with a healthcare provider at a health facility (See Supplementary Table 1c). Most women in Kano were interested in voice/text message and about half in social media. In Kano, four out of ten younger women and six out of ten older women were not interested in obtaining FP-related information through social media.

### Perceived importance and benefits of engaging with providers

#### Kenya

In Kenya, eight out of ten women said that engaging with a provider was “very/somewhat important” when starting or while using OCPs, ECs and self-injection. (See Supplementary File, Figure 1a). The three most reported perceived benefits of such engagements included learning about different methods (47%), learning how to use the selected method (42%), and having the provider recommend a method (42%). However, engaging with a provider was perceived by some women to waste time (54%), require unnecessary travel (39%), and increase cost (27%) (Table 2).

**Table 2.** Perceived benefits of engaging and not engaging with a provider^a^

	KENYA	LAGOS, NIGERIA	KANO, NIGERIA
	Weighted % (*n* = 9271)	Weighted % (*n* = 1291)	Weighted % (*n* = 1121)
**Reasons to engage with provider:^b^**			
Learn about different methods	46.6	30.3	25.9
Learn how to use selected method	42.4	29.3	24.4
Get recommendation	41.8	35.6	27.9
Get accurate information	40.7	30.9	28.4
Manage side effects	37.4	24.8	31.6
Close to home	33.9	10.5	30.0
Discreet/ confidential	29.5	11.0	34.1
Get quality products	27.0	18.2	20.2
Low cost	24.9	8.7	22.3
Already go to facility/Saves time	22.6	6.5	10.1
Method usually available	13.5	8.0	14.1
No benefit	2.4	11.4	6.4
**Reasons not to engage with provider:^c^**			
Saves time	54.1	25.8	47.9
No need to travel/less travel	39.2	13.5	27.9
Lower cost	26.7	11.3	20.5
Discreet/ confidential	24.0	9.2	20.4
I have more control	22.3	12.9	17.6
Flexible schedule/ Information when I want	18.7	5.2	6.6
No benefit	16.3	35.3	19.1
So I don't get infected while getting care	14.8	3.6	6.4
Do not feel comfortable	13.3	5.1	2.5

a Multiple responses are possible, spontaneous mention; responses with values of ≥10% in at least one site reported.

b “What do you see as the benefits of engaging with a provider when starting or while using oral contraceptive pills, emergency contraception, or injections that you could give yourself?”

c “What do you see as the benefits of NOT engaging with a provider when starting or while using oral contraceptive pills, emergency contraception, or injections that you could give yourself?”

#### Lagos

In Lagos, three quarters of women said that engaging with a provider was “very/somewhat important” when starting or while using OCPs, ECs and self-injection. (See Supplementary File, Figure 1b). The most common reasons to engage with a provider included getting the provider’s recommendation (36%), getting accurate information (31%), and learning about different methods (30%). However, engaging with a provider was perceived by some women to have no benefit (35%), waste time (26%), and require unnecessary travel (14%) (Table 2).

#### Kano

In Kano, nine out of ten women said that engaging with a provider was “very/somewhat important” when starting or while using OCPs, ECs and self-injection. (See Supplementary File, Figure 1c). The most cited reasons to engage with a provider included the discreet and confidential nature of their services (34%), management of side effects (32%), and the provider proximity to the woman’s home (30%). Reasons not to engage with a provider were to save time (48%), reduce travel (28%), and lower cost (21%) (Table 2).

### Preferred places of access for products amenable to self-administration

**Figure 2.**
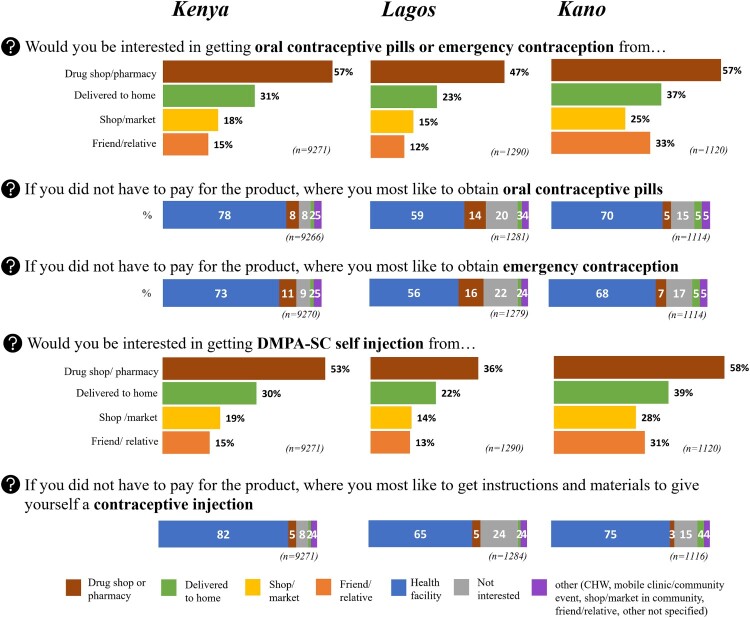
Interest in receiving method from various sources and preferred sources

We first asked women about their interest in receiving (1) OCPs or EC and (2) DMPA-SC from each of four places of access (Figure 2) other than a health facility – drug shop/pharmacy, delivered to their home, shop/market, and friend/relative. More respondents were interested in procuring contraceptive products from drug shops or pharmacies than from other places across methods and sites. Next, we asked women what their preferred place of access would be among these four places and more traditional options, including the health facility, community health workers, and outreach, if products were free. Across sites and products, most women indicated a preference for obtaining contraceptive products from health facilities.

#### Kenya

If they did not have to pay, about three-quarters of women would prefer to obtain OCPs, EC and materials to self-inject DMPA-SC from a health facility. When asked about their preferred place of access other than a health facility, 57% of Kenyan women showed interest in receiving OCPs or EC from drug shops or pharmacies while 53% preferred to obtain DMPA-SC self-injection from this source. Just under a third showed interest in having these products delivered to their homes (Figure 2).

We compared the preferred and actual places of access reported by current users of OCPs, EC, and injectables. In Kenya, the two most common sources of supply for OCP and injectables were the health facility (62% and 91%, respectively) and pharmacies (37% and 9%, respectively). Overall, 73% of OCP users and 93% of injectable users who supplied at the health facility reported the health facility as their preferred place of access. Among women who supplied at pharmacies, 52% of OCP users and 71% of injectable users said they would prefer getting their method from the health facility. Similar analyses for EC users in Kenya and for users of the three methods in Lagos and Kano are constrained by small numbers. However, they similarly point to some misalignment between actual and preferred sources among women supplying outside of health facilities (results not shown).

Multivariable models showed that across all three products, Kenyan women who were using a modern contraceptive method at the time of the survey, in wealthier households, and never married were more likely to report that they would prefer to obtain their method from a drug shop or pharmacy compared with the health facility (Table 3). Urban residents were also more likely to prefer receiving EC from a drug shop or pharmacy than from the health facility. However, women aged 25–49 years were less likely than those aged 15–24 years to prefer obtaining DMPA-SC self-injection from a drug shop or pharmacy compared to the health facility. Modern contraceptive users were more likely to prefer obtaining OCPs and DMPA-SC self-injection from other sources (shop/market, family/friends, delivered to home) compared with the health facility. Muslim women and those in the lower wealth quintile were less likely to prefer other sources of supply versus a health facility for DMPA-SC self-injection compared to Catholic women and women in the lowest wealth quintile, respectively. Similarly, women aged 25–49 years were less likely than those aged 15–24 years to prefer other sources to the health facility for OCPs and DMPA-SC self-injection.

**Table 3.** Multinomial logistic regression examining factors associated with preferred place of access, by product in Kenya

	Oral contraceptive pills (*n* = 8561)		Emergency Contraception (*n* = 8471)		DMPA-SC self-injection (*n* = 8563)	
	Drug shop/pharmacy vs. Health facility aRRR (95% CI)	Other places vs. health facility aRRR (95% CI)	Drug shop/pharmacy vs. Health facility aRRR (95% CI)	Other places vs. health facility aRRR (95% CI)	Drug shop/pharmacy vs. Health facility aRRR (95% CI)	Other places vs. health facility aRRR (95% CI)
**Education (ref: none/primary)**						
Post-primary/Secondary	1.04 (0.79–1.35) [*p* = 0.796]	1.04 (0.83–1.30) [*p* = 0.723]	1.00 (0.82–1.23) [*p* = 0.993]	1.05 (0.81–1.36) [*p* = 0.726]	0.96 (0.73–1.27) [*p* = 0.771]	1.00 (0.79–1.26) [*p* = 0.990]
College/University	1.25 (0.91–1.72) [*p* = 0.170]	1.31 (0.91–1.89) [*p* = 0.145]	1.09 (0.81–1.48) [*p* = 0.551]	1.34 (0.94–1.91) [*p* = 0.105]	1.25 (0.81–1.93) [*p* = 0.306]	1.41 (0.98–2.04) [*p* = 0.067]
**Marital status (ref: never married)**						
Married/living with partner	0.54 (0.41–0.69) [*p* < 0.001]	0.87 (0.64–1.18) [*p* = 0.376]	0.64 (0.51–0.80) [*p* < 0.001]	0.78 (0.58–1.05) [*p* = 0.100]	0.49 (0.35–0.70) [*p* < 0.001]	0.85 (0.62–1.15) [*p* = 0.290]
Divorced/separated/widowed	0.80 (0.54–1.20) [*p* = 0.285]	1.32 (0.86–2.04) [*p* = 0.207]	0.96 (0.70–1.33) [*p* = 0.815]	1.35 (0.90–2.01) [*p* = 0.142]	0.85 (0.53–1.34) [*p* = 0.516]	1.32 (0.88–1.98) [*p* = 0.177]
**Age (ref: 15–24)**						
25–49	0.78 (0.61–1.01) [*p* = 0.055]	0.65 (0.47–0.89) [*p* = 0.007]	0.83 (0.66–1.06) [*p* = 0.129]	0.79 (0.57–1.10) [*p* = 0.157]	0.69 (0.49–0.98) [*p* = 0.040]	0.66 (0.47–0.92) [*p* = 0.014]
**Religion (ref: Catholic)**						
Protestant	1.23 (0.90–1.67) [*p* = 0.193]	1.10 (0.78–1.55) [*p* = 0.597]	1.13 (0.87–1.48) [*p* = 0.368]	1.08 (0.78–1.52) [*p* = 0.632]	1.28 (0.88–1.87) [*p* = 0.191]	1.02 (0.65–1.58) [*p* = 0.941]
Islam	0.65 (0.30–1.43) [*p* = 0.285]	0.70 (0.33–1.49) [*p* = 0.356]	0.60 (0.33–1.11) [*p* = 0.103]	0.89 (0.39–2.00) [*p* = 0.771]	0.62 (0.22–1.75) [*p* = 0.368]	0.38 (0.15–0.97) [*p* = 0.042]
Other/unknown	1.14 (0.51–2.55) [*p* = 0.752]	1.40 (0.65–3.01) [*p* = 0.386]	1.18 (0.57–2.44) [*p* = 0.653]	1.31 (0.62–2.78) [*p* = 0.475]	0.83 (0.34–2.00) [*p* = 0.677]	1.16 (0.57–2.37) [*p* = 0.677]
**Geographic residence (ref: rural)**						
Urban	1.58 (0.98–2.54) [*p* = 0.059]	0.63 (0.33–1.20) [*p* = 0.160]	1.65 (1.08–2.53) [*p* = 0.020]	0.56 (0.29–1.05) [*p* = 0.072]	1.43 (0.76–2.71) [*p* = 0.268]	0.68 (0.32–1.45) [*p* = 0.309]
**Wealth quintile (ref: lowest quintile)**						
Lower quintile	1.09 (0.70–1.71) [*p* = 0.691]	0.65 (0.38–1.13) [*p* = 0.127]	1.04 (0.68–1.58) [*p* = 0.857]	0.66 (0.39–1.10) [*p* = 0.108]	1.14 (0.64–2.01) [*p* = 0.659]	0.55 (0.32–0.94) [*p* = 0.030]
Middle quintile	1.64 (1.10–2.43) [*p* = 0.014]	0.91 (0.49–1.72) [*p* = 0.780]	1.38 (0.97–1.98) [*p* = 0.077]	0.93 (0.51–1.71) [*p* = 0.824]	1.71 (1.07–2.74) [*p* = 0.026]	0.71 (0.36–1.41) [*p* = 0.330]
Higher quintile	1.95 (1.20–3.15) [*p* = 0.007]	0.91 (0.51–1.64) [*p* = 0.759]	1.65 (1.07–2.56) [*p* = 0.025]	0.86 (0.50–1.49) [*p* = 0.593]	1.91 (1.03–3.56) [*p* = 0.041]	0.74 (0.39–1.40) [*p* = 0.350]
Highest quintile	2.07 (1.26–3.40) [*p* = 0.004]	1.38 (0.67–2.83) [*p* = 0.383]	1.93 (1.23–3.04) [*p* = 0.005]	1.42 (0.72–2.79) [*p* = 0.313]	2.22 (1.18–4.18) [*p* = 0.014]	0.94 (0.39–2.28) [*p* = 0.893]
**Current use of any modern contraceptive method (ref: not using)**						
Currently using	1.66 (1.25–2.19) [*p* < 0.001]	1.35 (1.06–1.71) [*p* = 0.014]	1.62 (1.33–1.98) [*p* < 0.001]	1.24 (0.97–1.57) [*p* = 0.082]	1.75 (1.31–2.32) [*p* < 0.001]	1.39 (1.08–1.79) [*p* = 0.012]
**Empowerment tertiles (ref: lower)**						
Middle	1.13 (0.80–1.59) [*p* = 0.496]	0.88 (0.51–1.52) [*p* = 0.643]	1.20 (0.86–1.67) [*p* = 0.279]	0.93 (0.54–1.63) [*p* = 0.812]	1.04 (0.61–1.76) [*p* = 0.888]	0.77 (0.42–1.39) [*p* = 0.380]
High	0.99 (0.61–1.61) [*p* = 0.982]	0.83 (0.48–1.42) [*p* = 0.486]	1.00 (0.67–1.51) [*p* = 0.990]	0.84 (0.49–1.44) [*p* = 0.533]	1.02 (0.55–1.90) [*p* = 0.941]	0.58 (0.32–1.05) [*p* = 0.071]
**Fertility intentions (ref: desire to have a/another child)**						
Prefer no children/no more children	0.90 (0.71–1.14) [*p* = 0.377]	1.11 (0.78–1.58) [*p* = 0.576]	0.87 (0.70–1.07) [*p* = 0.188]	0.99 (0.69–1.42) [*p* = 0.948]	0.85 (0.61–1.18) [*p* = 0.330]	1.30 (0.86–1.96) [*p* = 0.220]
Don’t know/undecided/unable to be pregnant	1.04 (0.57–1.91) [*p* = 0.905]	0.90 (0.48–1.71) [*p* = 0.752]	1.25 (0.77–2.01) [*p* = 0.363]	1.00 (0.55–1.83) [*p* = 0.999]	0.86 (0.45–1.64) [*p* = 0.645]	1.34 (0.72–2.47) [*p* = 0.355]

aRRR =   adjusted relative risk ratio; CI =   confidence interval.

#### Lagos

If they did not have to pay, most women would prefer to obtain OCPs, EC and materials to self-inject DMPA-SC, respectively, from a health facility. In Lagos, 47% and 36% of women were interested in receiving OCPs/EC and DMPA-SC self-injection respectively from drug shops or pharmacies. Just under a fifth showed interest in having these products delivered to their homes. In Lagos, multivariable models revealed that current users of modern contraception and women who were not married or living with a partner were more likely to prefer obtaining OCPs from drug shops or pharmacies than from a health facility (Table 4). Women with no formal or primary education were more likely to prefer obtaining OCPs from other sources than from the health facility compared to women with higher education.

**Table 4.** Multinomial logistic regression examining factors associated with preferred place of access, by product in Lagos

	Oral contraceptive pills (*n* = 971)		Emergency Contraception (*n* = 948)		DMPA-SC self-injection (*n* = 923)	
	Drug shop/pharmacy vs. Health facility aRRR (95% CI)	Other places vs. health facility aRRR (95% CI)	Drug shop/pharmacy vs. Health facility aRRR (95% CI)	Other places vs. health facility aRRR (95% CI)	Drug shop/pharmacy vs. Health facility aRRR (95% CI)	Other places vs. health facility aRRR (95% CI)
**Education (ref: none/primary)**						
Secondary	1.15 (0.41–3.25) [*p* = 0.782]	0.63 (0.37–1.09) [*p* = 0.097]	1.12 (0.39–3.19) [*p* = 0.836]	0.41 (0.14–1.20) [*p* = 0.101]	1.25 (0.46–3.45) [*p* = 0.656]	0.65 (0.36–1.20) [*p* = 0.168]
Higher	0.92 (0.24–3.54) [*p* = 0.901]	0.45 (0.22–0.94) [*p* = 0.034]	1.04 (0.29–3.80) [*p* = 0.948]	0.40 (0.15–1.07) [*p* = 0.066]	1.40 (0.39–5.00) [*p* = 0.596]	0.43 (0.18–1.04) [*p* = 0.062]
**Marital status (ref: never married/divorced/separated/widowed)**						
Married/living with partner	0.45 (0.28–0.74) [*p* = 0.002]	0.80 (0.34–1.88) [*p* = 0.599]	0.63 (0.39–1.01) [*p* = 0.056]	0.65 (0.34–1.24) [*p* = 0.186]	0.65 (0.33–1.28) [*p* = 0.208]	0.75 (0.36–1.55) [*p* = 0.432]
**Age (ref: 15–24)**						
25–49	1.26 (0.77–2.04) [*p* = 0.350]	1.05 (0.47–2.36) [*p* = 0.901]	1.38 (0.79–2.43) [*p* = 0.256]	1.14 (0.62–2.10) [*p* = 0.669]	0.78 (0.34–1.76) [*p* = 0.540]	1.17 (0.58–2.33) [*p* = 0.656]
**Wealth tertile (ref: lowest tertile)**						
Middle tertile	1.80 (0.84–3.85) [*p* = 0.125]	0.77 (0.36–1.65) [*p* = 0.499]	1.73 (0.83–3.63) [*p* = 0.145]	0.59 (0.29–1.19) [*p* = 0.135]	1.05 (0.42–2.64) [*p* = 0.916]	0.72 (0.34–1.50) [*p* = 0.369]
Highest tertile	2.55 (0.96–6.76) [*p* = 0.061]	0.50 (0.24–1.08) [*p* = 0.078]	2.14 (0.88–5.19) [*p* = 0.092]	0.50 (0.20–1.21) [*p* = 0.121]	1.14 (0.45–2.86) [*p* = 0.778]	0.44 (0.19–1.04) [*p* = 0.060]
**Current use of any modern contraceptive method (ref: not using)**						
Currently using	1.76 (1.13–2.74) [*p* = 0.013]	0.76 (0.39–1.49) [*p* = 0.417]	1.51 (1.00–2.27) [*p* = 0.050]	0.75 (0.41–1.38) [*p* = 0.355]	1.37 (0.81–2.32) [*p* = 0.241]	1.01 (0.55–1.85) [*p* = 0.979]
**Empowerment tertiles (ref: lower)**						
Middle	0.78 (0.38–1.61) [*p* = 0.499]	1.87 (0.81–4.32) [*p* = 0.142]	0.70 (0.36–1.33) [*p* = 0.268]	1.82 (0.96–3.47) [*p* = 0.068]	1.07 (0.50–2.28) [*p* = 0.855]	1.63 (0.77–3.45) [*p* = 0.194]
High	0.78 (0.40–1.53) [*p* = 0.460]	0.86 (0.40–1.85) [*p* = 0.688]	0.85 (0.49–1.50) [*p* = 0.578]	0.76 (0.35–1.67) [*p* = 0.484]	0.64 (0.32–1.28) [*p* = 0.204]	1.15 (0.48–2.76) [*p* = 0.752]
**Fertility intentions (ref: desire to have a/another child)**						
Prefer no children/no more children	1.31 (0.60–2.89) [*p* = 0.495]	2.32 (0.83–6.49) [*p* = 0.105]	0.74 (0.27–2.02) [*p* = 0.553]	1.53 (0.41–5.66) [*p* = 0.519]	1.98 (0.63–6.21) [*p* = 0.237]	1.67 (0.51–5.44) [*p* = 0.389]
Don’t know/undecided/unable to be pregnant	0.84 (0.52–1.38) [*p* = 0.495]	1.05 (0.52–2.16) [*p* = 0.883]	0.71 (0.43–1.18) [*p* = 0.187]	1.07 (0.51–2.25) [*p* = 0.863]	0.87 (0.44–1.71) [*p* = 0.681]	1.24 (0.60–2.56) [*p* = 0.547]

aRRR = adjusted relative risk ratio; CI = confidence interval.

#### Kano

If they did not have to pay, a majority of women would prefer to obtain OCPs (70%), EC (68%) and materials to self-inject DMPA-SC (75%) respectively from a health facility, and 57–58% from drug shops or pharmacies. More than one-third of women were interested in having OCPs/EC and DMPA-SC self-injection delivered to their homes. In addition, approximately one third of participants were interested in obtaining products from a friend or relative, and about a quarter showed interest in procuring products at shops or markets. In Kano, multivariable models showed that women with secondary/higher education, compared to those with less education, were more likely to prefer drug shops/pharmacies over health facilities as their source of OCPs and EC (Table 5). Women who were unsure of their fertility intentions were more likely than those still wanting children to prefer sourcing OCPs from drug shops or pharmacies rather than from the health facility, while those who wanted no more children preferred to source DMPA-SC self-injection from drug shops or pharmacies over the health facility. Women who wanted no more children were more likely than those still wanting children to prefer other places of access (shop/market, family/friends, delivered to homes) to the health facility for all three products. Additionally, women in the middle empowerment tertile were less likely than those in the lower tertile to prefer other places of access over the health facility for OCPs and EC.

**Table 5.** Multinomial logistic regression examining factors associated with preferred place of access, by product in Kano

	Oral contraceptive pills (*n* = 786)		Emergency contraception (*n* = 758)		DMPA-SC self-injection (*n* = 792)	
	Drug shop/pharmacy vs. Health facility aRRR (95% CI)	Other places vs. health facility aRRR (95% CI)	Drug shop/pharmacy vs. Health facility aRRR (95% CI)	Other places vs. health facility aRRR (95% CI)	Drug shop/pharmacy vs. Health facility aRRR (95% CI)	Other places vs. health facility aRRR (95% CI)
**Education (ref: none/primary)**						
Secondary/higher	4.21 (2.14–8.34) [*p* < 0.001]	1.10 (0.61–1.99) [*p* = 0.740]	2.43 (1.16–5.09) [*p* = 0.021]	1.17 (0.60–2.30) [*p* = 0.630]	2.87 (0.95–8.69) [*p* = 0.061]	1.00 (0.24–4.22) [*p* = 0.997]
**Marital status (ref: never married/divorced/separated/widowed)**						
Married/living with partner	1.35 (0.27–4.74) [*p* = 0.703]	0.82 (0.28–2.41) [*p* = 0.704]	1.58 (0.33–7.61) [*p* = 0.552]	1.15 (0.37–3.62) [*p* = 0.798]	1.62 (0.37–7.09) [*p* = 0.506]	1.80 (0.66–4.94) [*p* = 0.242]
**Age (ref: 15–24)**						
25–49	0.67 (0.31–2.52) [*p* = 0.306]	0.67 (0.26–1.76) [*p* = 0.399]	0.59 (0.17–2.11) [*p* = 0.402]	0.68 (0.27–1.73) [*p* = 0.398]	0.55 (0.23–1.29) [*p* = 0.160]	0.43 (0.11–1.71) [*p* = 0.220]
**Wealth tertile (ref: lowest tertile)**						
Middle tertile	1.93 (0.55–6.99) [*p* = 0.291]	2.23 (0.62–8.06) [*p* = 0.208]	1.58 (0.40–6.19) [*p* = 0.495]	1.48 (0.33–6.68) [*p* = 0.594]	1.51 (0.37–6.11) [*p* = 0.548]	2.11 (0.37–11.92) [*p* = 0.382]
Highest tertile	1.36 (0.33–5.74) [*p* = 0.659]	3.75 (1.01–13.84) [*p* = 0.048]	1.74 (0.28–10.92) [*p* = 0.541]	2.92 (0.59–14.33) [*p* = 0.177]	2.00 (0.42–9.63) [*p* = 0.370]	2.64 (0.36–19.29) [*p* = 0.322]
**Current use of any modern contraceptive method (ref: not using)**						
Currently using	1.23 (0.38–3.97) [*p* = 0.717]	1.62 (0.58–4.52) [*p* = 0.340]	1.39 (0.45–4.28) [*p* = 0.551]	1.11 (0.32–3.79) [*p* = 0.866]	0.60 (0.20–1.79) [*p* = 0.345]	1.59 (0.42–5.88) [*p* = 0.472]
**Empowerment tertiles (ref: lower)**						
Middle	1.06 (0.57–2.00) [*p* = 0.848]	0.50 (0.31–0.81) [*p* = 0.007]	0.74 (0.40–1.37) [*p* = 0.323]	0.60 (0.37–0.95) [*p* = 0.030]	2.40 (0.94–6.14) [*p* = 0.065]	1.48 (0.86–2.56) [*p* = 0.151]
High	0.53 (0.25–1.14) [*p* = 0.110]	0.72 (0.27–1.97) [*p* = 0.508]	0.52 (0.22–1.23) [*p* = 0.129]	0.95 (0.34–2.67) [*p* = 0.917]	1.04 (0.28–3.78) [*p* = 0.956]	1.36 (0.65–2.83) [*p* = 0.397]
**Fertility intentions (ref: desire to have a/another child)**						
Prefer no children/no more children	1.74 (0.90–3.42) [*p* = 0.095]	2.56 (1.46–4.48) [*p* = 0.002]	1.46 (0.73–2.92) [*p* = 0.274]	2.23 (1.33–3.74) [*p* = 0.004]	3.22 (1.09–9.49) [*p* = 0.035]	3.38 (1.69–6.78) [*p* = 0.001]
Don’t know/undecided/unable to be pregnant	3.48 (1.34–8.98) [*p* = 0.013]	1.08 (0.58–2.01) [*p* = 0.801]	3.51 (0.99–12.41) [*p* = 0.052]	1.03 (0.48–2.21) [*p* = 0.937]	0.60 (0.17–2.13) [*p* = 0.414]	1.16 (0.44–3.11) [*p* = 0.751]

aRRR = adjusted relative risk ratio; CI = confidence interval.

Additional analyses (results not shown) revealed that across all three sites and all three methods, while the majority of women who were not currently using a modern contraceptive method preferred the health facility as their source, if they were to use a method, 3–12% would prefer to obtain their method from a drug shop, pharmacy, or other source.

## Discussion

These data, coming from a mini-module added to nationally or state representative PMA surveys in Kenya and in Kano and Lagos states in Nigeria, provide important information on women’s interest in a range of self-care interventions and their preferences for accessing them across three uniquely different contexts. Information on the perspectives of potential end-users is important to help decision-makers prioritise how to best incorporate self-care into FP policies and programmes in a way that is aligned with women’s preferences and upholds their rights. Overall, our findings show high interest in mobile access to information on a range of topics related to fertility, pregnancy and side effect management. Results confirm the relevance of drug shops and pharmacies as access points for over-the-counter methods amenable to self-administration and point to some openness to emerging strategies, like home delivery of contraceptive products. Women (potential clients) also note a strong preference for engaging with providers when obtaining or while using methods amenable to self-administration.

This evidence is timely in Kenya and Nigeria where self-care guidance documents have been launched. In Nigeria, the National Guideline on Self-care for Sexual, Reproductive and Maternal Health was launched on March 9, 2022 as part of the federal government’s commitment to achieving universal health coverage.^44^ Even though 20 of 24 WHO self-care recommendations were adopted in Nigeria, two recommendations were modified, including one related to OCP supply. Specifically, the guideline changed WHO’s recommendation to “provide up to one year’s supply of [oral contraceptive] pills” to “provide three months’ supply of [oral contraceptive] pills in public outlets, and up to one year’s supply over the counter in private outlets”.^24^ In both Nigeria and Kenya, OCPs and EC are available over the counter without a doctor’s prescription. In Kenya, the Ministry of Health launched the National Guideline for Self-care in Reproductive Health in January 2023. The guideline is in line with WHO’s 2022 revised guideline and was introduced to “enable health care providers to support safe and contextualised self-care in reproductive health” and to contribute to the country’s quest to achieve universal health coverage.^25^

Mobile access to information may prove to be important as one of multiple strategies in achieving universal health coverage. Our findings support the use of digital strategies for sharing reproductive health information. Although many platforms focus on the provision of information on contraceptive options, our results show there is a strong appetite for a wider range of information inclusive of fertility and side effect management. Despite the growth of client-facing digital platforms, a recent review noted that side effects still tend to be under-represented in their content.^45^ The range of digital technologies used to support the provision of reproductive health and FP information has expanded in recent years with an increased reliance on chatbots. Our results suggest that text messages appeal to many women across the three sites. Openness to interacting with social media as a source of information was more mixed and varied across sites, but the appeal of social media was consistently greater among younger women. More women in Lagos were interested in this approach compared to other settings, possibly due to the more urban nature of the sample or higher levels of pre-existing use of social media. Importantly, our findings show that interest in mobile access to information exceeds the current levels of receiving such information. Across sites, over 88% of participants in the mini-module expressed interest in voice/text messages and over 49% in social media. In the main PMA questionnaire, 3–18% of women reported receiving a voice or text message about FP on a mobile phone in the 12 months preceding the survey, and 4–37%, seeing anything on social media about FP. Despite the appeal of obtaining FP information through digital platforms, this source of information is not always regulated. For instance, social media is available for everyone to use but these digital platforms do not always provide accurate information for users as writers can claim expertise they do not possess. Ethical concerns have been raised related to the credibility of the content writers on unregulated sites that young people moderate and frequent. Most social media sites have privacy controls but not all users actually restrict access to their profiles, thus raising concerns about the privacy and confidentiality of social media platform users.^21^

Survey data,^15^ including from the main PMA questionnaire in 2021/2022 to which our mini-module was added, shows that drug shops and pharmacies are important sources of supply for OCPs and EC in Kenya and Nigeria. Our findings on interest in and preferences for confirm this for these two methods, but also for DMPA-SC, whose provision is still limited in the two settings. These common private sector sources are a promising high-impact practice in FP service delivery.^46^ However, our results also underscore the importance of linking self-care interventions to the formal health systems. We found that health facilities were the preferred source for obtaining these products across sites. A study conducted in urban Kenya and Nigeria also reported that 44% of women obtained OCPs from pharmacies and drug shops and 56% from health facilities.^14^ Moreover, women highly valued engagement with healthcare providers when starting or while using methods, largely as a way to obtain information in Kenya and Lagos, but also for convenience and privacy in Kano.

Findings provide some evidence of misalignment between actual and preferred place of access. Over half of the women who obtained OCPs from outside the facility in Kenya would have preferred to obtain the method from the facility, suggesting that women may be using these other access points because they are unable, possibly as a result of distance, required time commitment, or stockouts, to obtain their method of choice from their preferred source, the health facility. A question requiring further research is whether self-care for contraception is based on choice or due to convenience, cost or other factors.^14^ Even though the findings of this study suggest that non-facility access points hold potential, both for women who are modern contraceptive users and those not currently using contraception, this must be examined with caution given our findings that suggest that the use of these non-facility sources may not always be by choice.

Considering health worker shortages and other systemic challenges, especially in public health facilities, enabling the potential for greater use of non-health-facility sources warrants attention to expand the range of options women have. While the provision of OCPs and EC through drug shops and pharmacies is not new and already successful, the availability of DMPA-SC is more limited. More could also be done to support access through these channels. Taken together, findings on mobile access to information and preferences for accessing products deserve further exploration on how mobile technology may be used to extend some of the informational benefits noted from engaging with providers and open windows of opportunities for women to procure products through these outlets that may offer greater convenience. The Pharmacists Council that regulates the activities and practice of both pharmacists and drug shop owners in Nigeria and the Pharmacy and Poisons Board in Kenya are also positioned to put in place more stringent measures to ensure that only high-quality products are sold in all outlets with specific contraceptive training for those who dispense contraceptives. Interestingly, our results also point to potentially sizable acceptability of other emerging options such as home delivery of contraceptive products. Some digital tools have started to incorporate online marketplaces for health products, including contraceptives, for home delivery.^47^ This finding highlights potential windows of opportunities for these approaches.

Despite the predominant preference for health facilities, multivariable analyses revealed some interesting differences between subgroups of potential end users. Urban and wealthier women in Kenya and more educated women in Kano are more likely than women in rural areas and those who are poorer or less educated to be interested in some non-facility-based venues to access some products. In Kenya, ever-married women and women aged 25–49 years were less likely than their never-married and younger counterparts to prefer to obtain their method from non-facility access points over health facilities. This latter finding aligns with a study conducted in urban Nigeria and Kenya,^14^ as well as findings from a multi-country analysis exploring the characteristics of clients of pharmacies and drug shops across 12 SSA countries, using data from the Demographic and Health Surveys.^48^ Existing literature suggests that drug shops and pharmacies appeal to a heterogenous group of new or continuing contraceptive users. These include more educated, urban, wealthier women who lack the time to wait in traditional health facilities^14^ as well as poor, urban or rural women who appreciate the privacy and confidentiality these non-facility access points provide. Another group of women who prefer non-facility access points for their self-care amenable contraceptives are single, young women who may face stigma if they go to traditional health facilities in many SSA countries where chastity before marriage is a widely upheld moral expectation.^14,49–51^

Our study revealed a significant relationship between contraceptive empowerment and the preferred place of access to contraceptive self-care products (OCPs and EC) in Kano, but not in Lagos or Kenya. Contraceptive empowerment in this study reflects the existence and exercise of contraceptive choice. Women in the middle contraceptive empowerment tertile were more likely to report a preference for accessing contraceptive self-care products in health facilities rather than other places of access (i.e. community health worker, mobile clinic, shop/market, friend/relative) when compared with women in the lower tertile. In a very conservative setting like Kano, this finding suggests women’s willingness to overtly use modern contraception. As frameworks and measurement of related FP concepts evolve, increasing emphasis is being placed on a rights-based framing and on the need to acknowledge what women want. While this is still an evolving area,^10^ our findings provide some insight into the relationship between contraceptive empowerment and self-care for FP behaviours and how this may differ by sociocultural context.

### Strengths and limitations

The addition of a mini-module to the PMA surveys in Kenya, Lagos and Kano allowed capturing the perspectives of large samples of women on a range of self-care interventions. Nigeria data are representative at the state level, and not generalisable to the whole country. Thus, potential variations in preferences among women living in other areas of the country need to be considered to inform national policy decisions. Space constraints did not allow us to examine preferences for accessing methods at initiation and at resupply separately; this area warrants more research. The limited number of questions and the quantitative approach restricted our ability to collect additional information to better understand and contextualise results. The predictive validity of preference data is also unknown. Places of access included both actual and hypothetical options; it is possible that some women may have been more inclined to express interest in scenarios they knew corresponded to the current environment. However, we believe that this would be a minority and would not bias the results in any significant way.

## Conclusion

Our results provide important information from the perspectives of women across three different contexts to help decision-makers set priorities for expanding the scope of self-care interventions within their context. Results highlight opportunities for the provision of products amenable to self-administration through health facilities, as well as outside of the traditional health system – notably through drug shops and pharmacies, a promising high-impact practice^46^ – and through adjunct use of digital health technologies. While these findings were broadly consistent across Kenya and Kano and Lagos in Nigeria, there were also some nuances across contexts and population subgroups, reinforcing the need to tailor programming to the specificities of each setting and to offer multipronged tailored interventions that offer a range of touchpoints with the health system to enable choice.

## Supplementary Material

Supplemental Figure 1a

Supplemental Figure 1b

Supplemental Figure 1c

Supplemental Table 1b

Supplemental Table 1a Kenya

Supplemental Table 1c

## Data Availability

Data can be accessed by submitting a request to https://www.pmadata.org/data/request-access-datasets.

## Provenance statement

This article was not commissioned and went through external peer review.
