# Supplementary figures and images for: Interests and preferences regarding family planning self-care interventions: cross-sectional surveys with Kenyan and Nigerian women
Source: Sex Reprod Health Matters. 2026 Jun 1;33(1):2681342. doi: 10.1080/26410397.2026.2681342 (PMC13295108; doi:10.1080/26410397.2026.2681342)

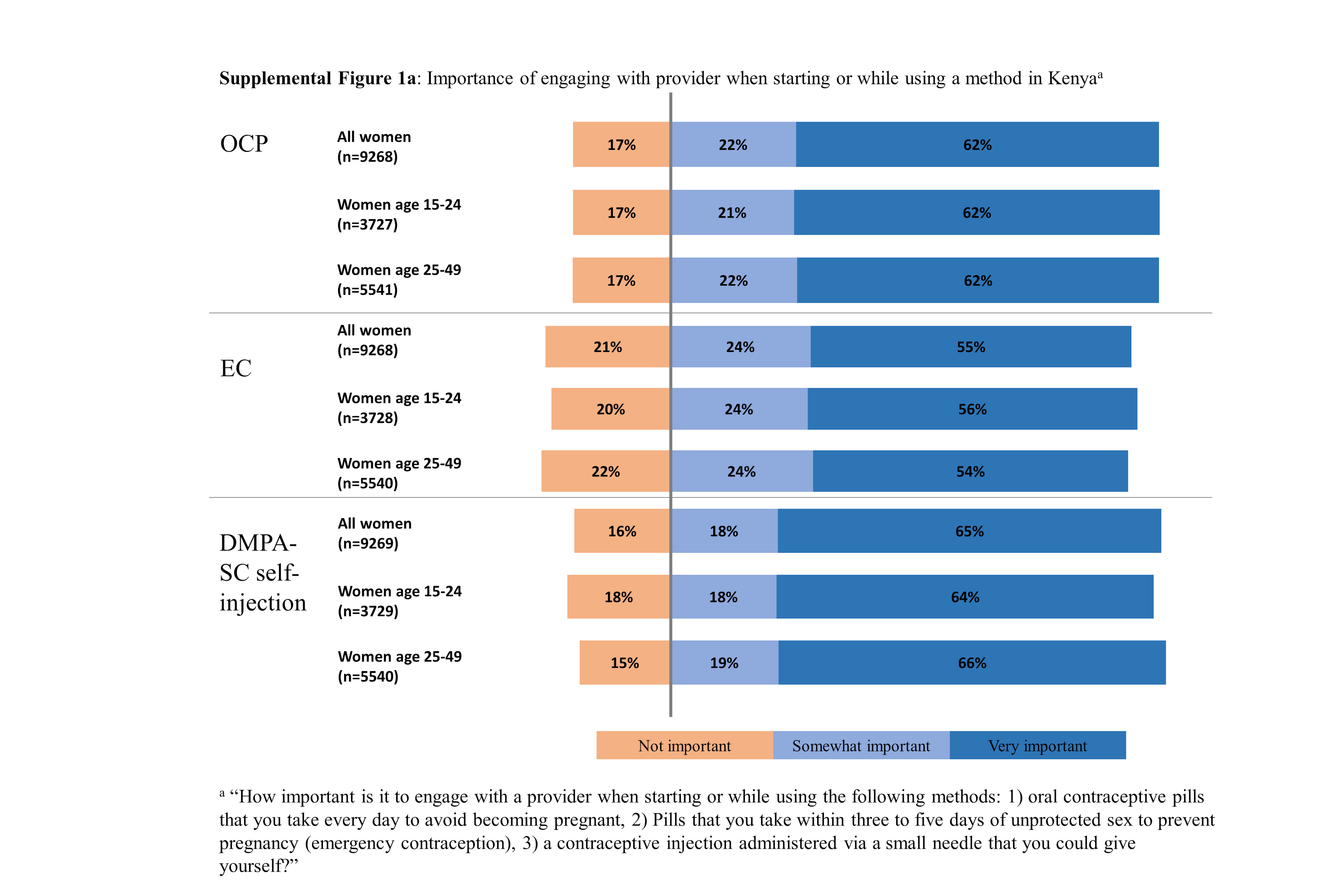

Supplement: Supplemental Figure 1a [file ZRHM_A_2681342_SM3589.tif]

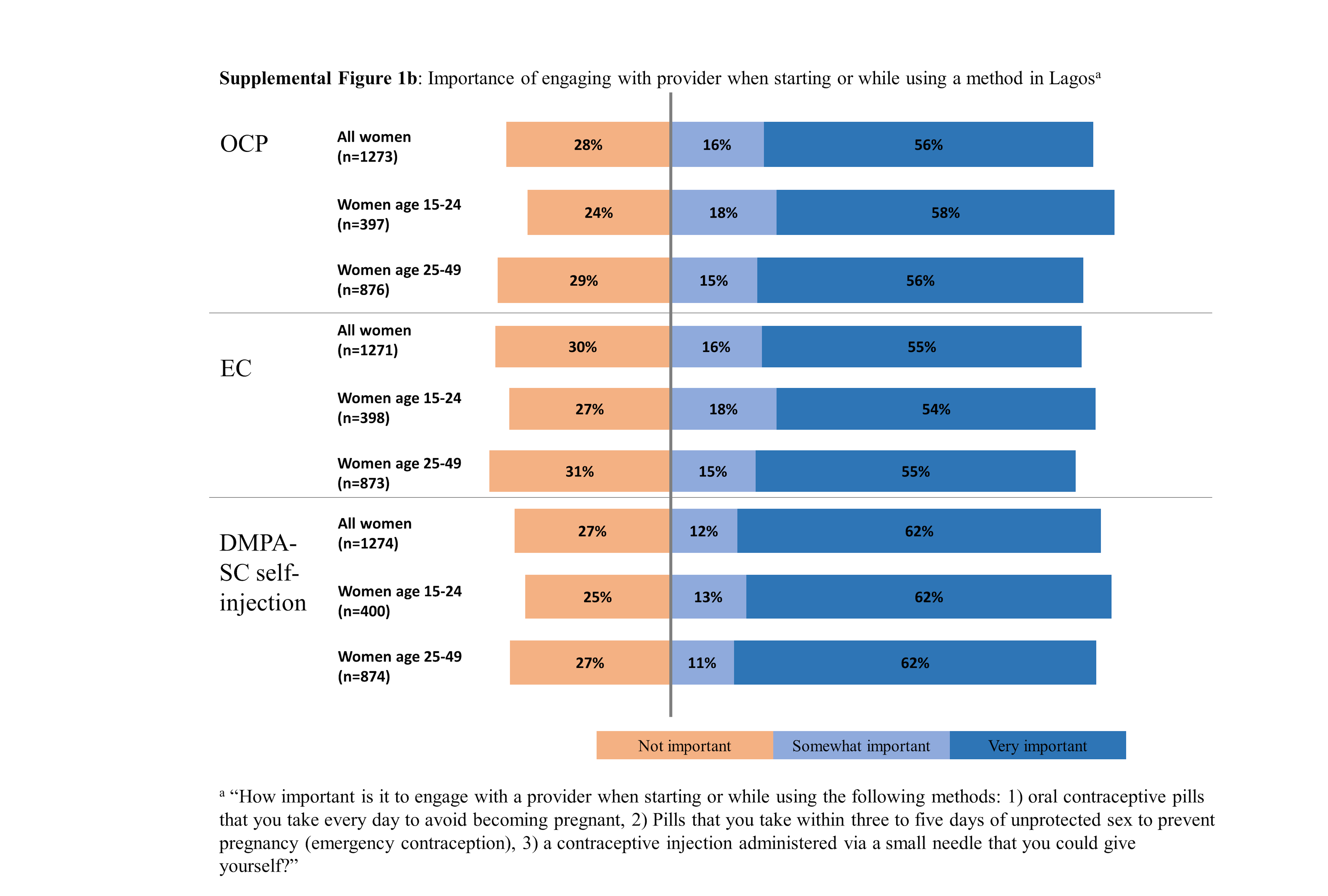

Supplement: Supplemental Figure 1b [file ZRHM_A_2681342_SM3588.tif]

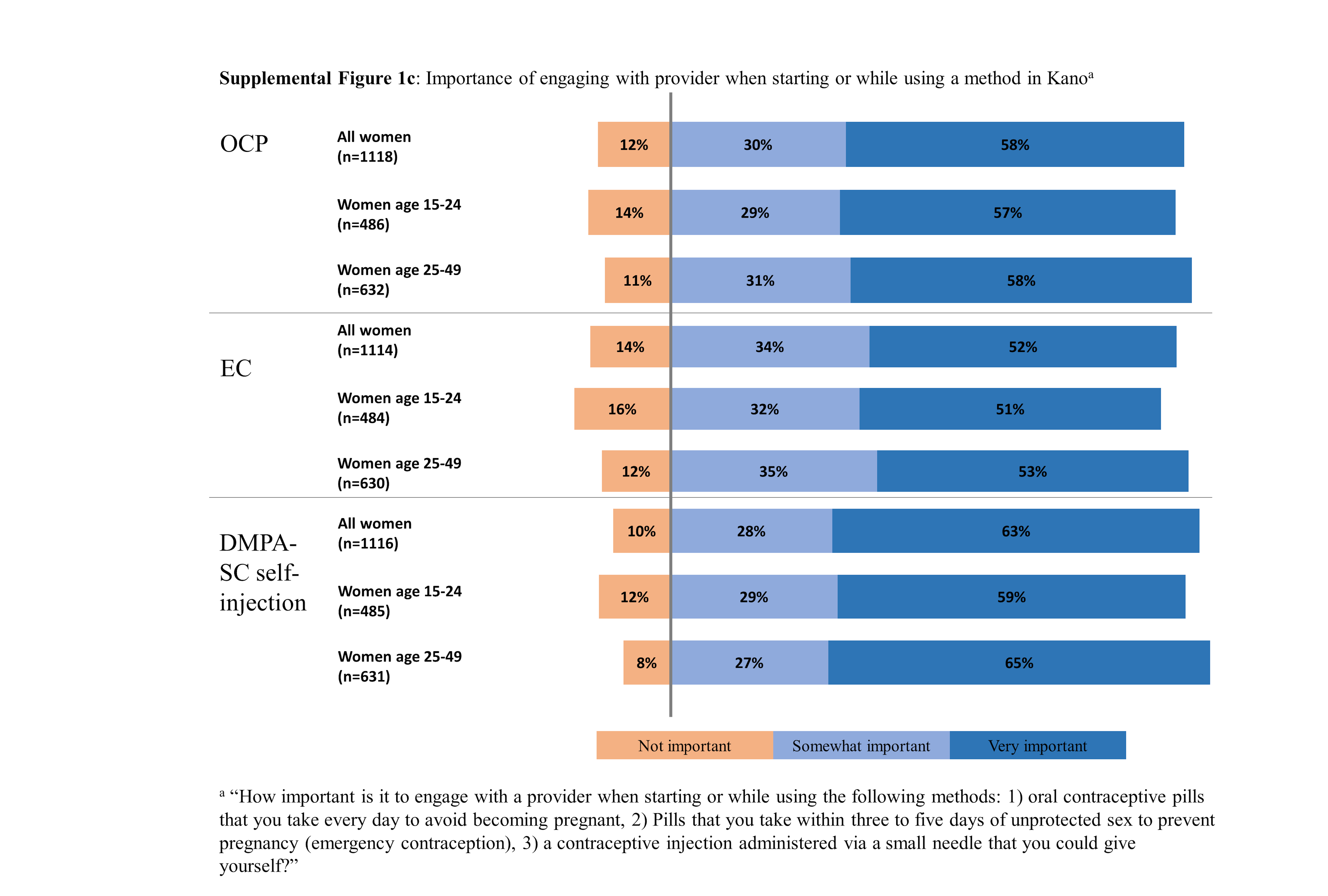

Supplement: Supplemental Figure 1c [file ZRHM_A_2681342_SM3587.tif]
