# Supplementary material for: Interests and preferences regarding family planning self-care interventions: cross-sectional surveys with Kenyan and Nigerian women
Source: Sex Reprod Health Matters. 2026 Jun 1;33(1):2681342. doi: 10.1080/26410397.2026.2681342 (PMC13295108; doi:10.1080/26410397.2026.2681342)
Supplement: Supplemental Table 1b [file ZRHM_A_2681342_SM3586.docx]

**Supplemental Table 1b: Types of information and sources of information women and girls are interested in within the context of self-care in Lagos**

|  | **TOTAL, %^^^** |  | **AGE 15-24, %^^^** |  | **AGE 25-49, %^^^** |  | **χ^2^ (p-value)** |
| --- | --- | --- | --- | --- | --- | --- | --- |
|  |  |  |  |  |  |  |  |
| **Type of information interested in accessing on their own:^a^** | **100.0**  **(N=1291)** |  | **31.7**  **(N=409)** |  | **68.3**  **(N=882)** |  |  |
| Managing contraceptive-induced menstrual changes |  |  |  |  |  |  | 0.29 (0.676) |
| Interested | 74.3 |  | 73.3 |  | 74.7 |  |  |
| Not interested/already have info | 25.7 |  | 26.7 |  | 25.3 |  |  |
| Managing side effects |  |  |  |  |  |  | 0.02 (0.911) |
| Interested | 72.2 |  | 72.0 |  | 72.3 |  |  |
| Not interested/already have info | 27.8 |  | 28.0 |  | 27.7 |  |  |
| Identifying fertile days |  |  |  |  |  |  | 0.30 (0.597) |
| Interested | 74.0 |  | 75.0 |  | 73.5 |  |  |
| Not interested/already have info | 26.0 |  | 25.0 |  | 26.5 |  |  |
| Confirming pregnancy |  |  |  |  |  |  | 1.96 (0.294) |
| Interested | 69.9 |  | 67.2 |  | 71.1 |  |  |
| Not interested/already have info | 30.1 |  | 32.8 |  | 28.9 |  |  |
| Assessing return to fertility postpartum |  |  |  |  |  |  | 0.35 (0.629) |
| Interested | 68.9 |  | 70.0 |  | 68.4 |  |  |
| Not interested/already have info | 31.1 |  | 30.0 |  | 31.6 |  |  |
|  |  |  |  |  |  |  |  |
| **Among women interested in accessing information on their own, preferred source of information:** | **100.0**  **(N=1041)** |  | **31.4**  **(N=327)** |  | **68.6**  **(N=714)** |  |  |
| Voice or text message on mobile phone^b^ |  |  |  |  |  |  | 5.38 (0.035) |
| Interested | 87.6 |  | 84.0 |  | 89.1 |  |  |
| Not interested | 12.4 |  | 16.0 |  | 10.9 |  |  |
| Social media^c^ |  |  |  |  |  |  | 1.95 (0.215) |
| Interested | 71.6 |  | 74.5 |  | 70.3 |  |  |
| Not interested | 28.4 |  | 25.5 |  | 29.7 |  |  |

Due to small amounts of missing data, not all denominators match the table headings

^^^Frequencies are unadjusted; percentages are adjusted for sampling weights

^a^ The introduction to the questions included an explanation that “on your own” means “without necessarily having to access or speak with a healthcare provider at a health facility.”

^b^ Would you be interested in receiving a voice or text message with this type of information on a mobile phone?

^c^ Would you be interested in receiving this type of information on social media such as Facebook, Viber, Twitter, WhatsApp or others?
