# Supplementary material for: Interests and preferences regarding family planning self-care interventions: cross-sectional surveys with Kenyan and Nigerian women
Source: Sex Reprod Health Matters. 2026 Jun 1;33(1):2681342. doi: 10.1080/26410397.2026.2681342 (PMC13295108; doi:10.1080/26410397.2026.2681342)
Supplement: Supplemental Table 1a Kenya [file ZRHM_A_2681342_SM3585.docx]

**Supplemental Table 1a: Types of information and sources of information women and girls are interested in within the context of self-care in Kenya**

|  | **TOTAL, %^^^** |  | **AGE 15-24, %^^^** |  | **AGE 25-49, %^^^** |  | **χ^2^ (p-value)** |
| --- | --- | --- | --- | --- | --- | --- | --- |
|  |  |  |  |  |  |  |  |
| **Type of information interested in accessing on their own:^a^** | **100.0**  **(N=9271)** |  | **40.2**  **(N=3730)** |  | **59.8**  **(N=5541)** |  |  |
| Managing contraceptive-induced menstrual changes |  |  |  |  |  |  | 4.03 (0.093) |
| Interested | 85.4 |  | 84.5 |  | 86.0 |  |  |
| Not interested/already have info | 14.6 |  | 15.5 |  | 14.0 |  |  |
| Managing side effects |  |  |  |  |  |  | 11.46 (0.011) |
| Interested | 85.3 |  | 83.8 |  | 86.3 |  |  |
| Not interested/already have info | 14.7 |  | 16.2 |  | 13.7 |  |  |
| Identifying fertile days |  |  |  |  |  |  | 7.10 (0.033) |
| Interested | 83.9 |  | 85.1 |  | 83.1 |  |  |
| Not interested/already have info | 16.1 |  | 14.9 |  | 16.9 |  |  |
| Confirming pregnancy |  |  |  |  |  |  | 1.96 (0.295) |
| Interested | 82.6 |  | 83.3 |  | 82.2 |  |  |
| Not interested/already have info | 17.4 |  | 16.7 |  | 17.8 |  |  |
| Assessing return to fertility postpartum |  |  |  |  |  |  | 0.99 (0.450) |
| Interested | 81.0 |  | 81.5 |  | 80.7 |  |  |
| Not interested/already have info | 19.0 |  | 18.5 |  | 19.3 |  |  |
|  |  |  |  |  |  |  |  |
| **Among women interested in accessing information on their own, preferred source of information:** | **100.0**  **(N=8348)** |  | **40.2**  **(N=3353)** |  | **59.8**  **(N=4995)** |  |  |
| Voice or text message on mobile phone^b^ |  |  |  |  |  |  | 30.29 (<0.001) |
| Interested | 90.4 |  | 88.3 |  | 91.9 |  |  |
| Not interested/already do | 9.6 |  | 11.7 |  | 8.1 |  |  |
| Social media^c^ |  |  |  |  |  |  | 111.46 (<0.001) |
| Interested | 56.2 |  | 63.1 |  | 51.5 |  |  |
| Not interested/already do | 43.8 |  | 36.9 |  | 48.5 |  |  |

Due to small amounts of missing data, not all denominators match the table headings

^^^Frequencies are unadjusted; percentages are adjusted for sampling weights

^a^ The introduction to the questions included an explanation that “on your own” means “without necessarily having to access or speak with a healthcare provider at a health facility.”

^b^ Would you be interested in receiving a voice or text message with this type of information on a mobile phone?

^c^ Would you be interested in receiving this type of information on social media such as Facebook, Viber, Twitter, WhatsApp or others?
