# Supplementary material for: Interests and preferences regarding family planning self-care interventions: cross-sectional surveys with Kenyan and Nigerian women
Source: Sex Reprod Health Matters. 2026 Jun 1;33(1):2681342. doi: 10.1080/26410397.2026.2681342 (PMC13295108; doi:10.1080/26410397.2026.2681342)
Supplement: Supplemental Table 1c [file ZRHM_A_2681342_SM3584.docx]

**Supplemental Table 1c: Types of information and sources of information women and girls are interested in within the context of self-care in Kano**

|  | **TOTAL, %^^^** |  | **AGE 15-24, %^^^** |  | **AGE 25-49, %^^^** |  | **χ^2^ (p-value)** |
| --- | --- | --- | --- | --- | --- | --- | --- |
|  |  |  |  |  |  |  |  |
| **Type of information interested in accessing on their own:^a^** | **100.0**  **(N=1121)** |  | **43.6**  **(N=489)** |  | **56.4**  **(N=632)** |  |  |
| Managing contraceptive-induced menstrual changes |  |  |  |  |  |  | 6.60 (0.078) |
| Interested | 84.6 |  | 81.3 |  | 87.0 |  |  |
| Not interested/already have info | 15.4 |  | 18.7 |  | 13.0 |  |  |
| Managing side effects |  |  |  |  |  |  | 10.83 (0.013) |
| Interested | 85.2 |  | 81.2 |  | 88.2 |  |  |
| Not interested/already have info | 14.8 |  | 18.8 |  | 11.8 |  |  |
| Identifying fertile days |  |  |  |  |  |  | 7.41 (0.059) |
| Interested | 84.7 |  | 81.3 |  | 87.2 |  |  |
| Not interested/already have info | 15.3 |  | 18.7 |  | 12.8 |  |  |
| Confirming pregnancy |  |  |  |  |  |  | 15.95 (0.008) |
| Interested | 82.6 |  | 77.4 |  | 86.5 |  |  |
| Not interested/already have info | 17.4 |  | 22.6 |  | 13.5 |  |  |
| Assessing return to fertility postpartum |  |  |  |  |  |  | 17.65 (0.009) |
| Interested | 83.8 |  | 78.4 |  | 87.8 |  |  |
| Not interested/already have info | 16.2 |  | 21.6 |  | 12.2 |  |  |
|  |  |  |  |  |  |  |  |
| **Among women interested in accessing information on their own, preferred source of information:** | **100.0**  **(N=954)** |  | **41.0**  **(N=391)** |  | **59.0**  **(N=563)** |  |  |
| Voice or text message on mobile phone^b^ |  |  |  |  |  |  | 2.57 (0.130) |
| Interested | 90.0 |  | 91.9 |  | 88.7 |  |  |
| Not interested | 10.0 |  | 8.1 |  | 11.3 |  |  |
| Social media^c^ |  |  |  |  |  |  | 37.38 (<0.001) |
| Interested | 49.1 |  | 61.1 |  | 40.9 |  |  |
| Not interested | 50.9 |  | 38.9 |  | 59.1 |  |  |

Due to small amounts of missing data, not all denominators match the table headings

^^^Frequencies are unadjusted; percentages are adjusted for sampling weights

^a^ The introduction to the questions included an explanation that “on your own” means “without necessarily having to access or speak with a healthcare provider at a health facility.”

^b^ Would you be interested in receiving a voice or text message with this type of information on a mobile phone?

^c^ Would you be interested in receiving this type of information on social media such as Facebook, Viber, Twitter, WhatsApp or others?
